# The Dark Triad and the Detection of Parental Judicial Manipulators. Development of a Judicial Manipulation Scale

**DOI:** 10.3390/ijerph17082843

**Published:** 2020-04-21

**Authors:** Miguel Clemente, Dolores Padilla-Racero, Pablo Espinosa

**Affiliations:** Department of Psychology, Universidade da Coruña, 15071 A Coruña, Spain; miguel.clemente@udc.es (M.C.); dolores.padilla@udc.es (D.P.-R.)

**Keywords:** judicial behavior, children, manipulation, dark Triad, Machiavellianism, Narcissism, psychopathy

## Abstract

This research examines the relationship between dark triad and the use that some parents make of their children in order to attack the other parent after a couple break-up. We examined whether parents who are willing to lie about issues concerning the other parent and their children during a couple break-up process show higher levels of dark triad traits. Across two different samples of divorced participants (*N* = 1085 and *N* = 249), we measured dark triad traits and willingness to engage in judicial manipulation. The objective of this study was to build a judicial manipulation scale to measure willingness to lie and use children to harm the other parent that could be used in professional practice. Results show significant correlations for judicial manipulation and dark triad traits and confirm the psychometric properties of reliability and validity of a proposed scale. We found that dark triad traits are adequate indicators of judicial manipulation. We discuss the importance of the scale to help the judicial system to determine which parent is the most appropriate to be designated as the legal custodial parent.

## 1. Introduction

One of the current problems faced by contemporary society is the continuous increase of couple break-ups; hence, many children grow up under the protection of only one of their parents. When a couple that has children breaks up, it is difficult to be detach the children from the fight between the parents; what is even worse, the opposite may happen and the children become a weapon to attack the (ex-)partner. In line with these tenets, Gardner [1,2,3,4] created the concept of the parental alienation syndrome (PAS), which claims that often one of the parents (normally the mother, who tends to be the custodial parent) mentally manipulates children so they will not wish to have contact with the other parent (usually the father and non-custodial parent). Thus, the alienating parent induces the child to lie about the other parent harming them. Children would then start elaborating on these false claims and deluding themselves into believing that the other parent has actually harmed them. According to PAS, the alienating parent manages to convince the child that the other parent assaulted them physically. The goal of the manipulative parent is that the justice system intervenes to punish the ex-partner and to prevent contact between the child and the allegedly aggressive father. A problem with PAS assumptions is the belief that children routinely lie and are easy to manipulate against the noncustodial parent, making up claims of non-existent aggressions.

Sometimes, the accusation is of sexual abuse, which often cannot be medically proven, and this inability to provide evidence is taken as a confirmation of PAS. However, the fact that it cannot be proven does not mean it does not exist, and there are cases of children’s physically and sexually abused by their parents that do not show medical signs of the damage caused. In these cases, the only way to discover the existence of the abuse is by resorting to the testimony of the minor (if the child is old enough) or through the detection of some typical signs of abuse. Abusive parents may argue that it is an invention of the child, who has been convinced by the other parent to lie and invent non-existent aggressions. Therefore, they will state that the abuse is exerted by the parent who is allegedly manipulating the child. Subsequently, they will claim that the court must act to prevent it and protect the child, who is being manipulated and the parent the child refuses contact with. The existing evidence shows that complaints of abuse and, specifically, of sexual abuse are very rarely false [5,6,7,8,9,10] and that parents, specifically the guardian, normally do not invent aggressions that the child has not suffered nor do they instill any belief in the child. Hence, parental complaints are most times the result of actual child abuse and the Justice system runs the risk of failing to provide protection, based on a false argument that the minor is being manipulated by the custodial parent [6,11,12,13,14,15,16,17]. However, despite this evidence, some authors still support that accusations of maltreatment or abuse toward minors advocated by Gardner are frequent [18,19,20,21,22,23,24,25,26,27,28,29].

Although from a legal point of view, the topic (protection of minors) is of great relevance, it is no less relevant from a psychological perspective, because a variable—manipulation—is introduced in the parent-child relationship. Thus, from the perspective of psychology, it is important to determine whether parents offer affection and sincerity to their children, doing everything they can to educate them in positive values, or, in contrast, they don’t mind using their children to attack the other parent, often as vengeance for breaking up the relationship. Up to 75% of parents report difficulties in the relationship with their ex-partner [30], and custody conflicts, often accompanied with financial conflicts, are related to negative emotional, behavioral and academic effects on children [31], both externalizing and internalizing [32]. So judicial manipulation is harmful and devastating for the victim parent, but also extremely serious for the child. As stated above, the PAS, which focuses on the effect of manipulative behavior on the parents and the children, provides a popular approach to this issue [18,24,26,27,28,29]. However, other authors [13,14,33,34] argue that most assumptions derived from the PAS are questionable.

Rather than adopting the PAS approach, evaluating specific traits that predict manipulative behavior seems more promising. Narcissistic individuals have lower ethical standards in their pursuit of self-interest, and thus are more prone to manipulative behavior [35]. Machiavellianism is also a good indicator of lying in different situations [36] and dark traits of personality influence willingness to make false claims in legal settings [37]. Traits in the dark triad may be sound indicators of manipulative behavior against the ex-partner. In related domains, psychopathy [38] predicts divorce and undermines marital relationship functioning. High Narcissism is also related to conflicts over visitation and custody of children after divorce [39] and disengagement from children in the non-custodial parent after divorce litigation [40]. Currently, research has established that Machiavellianism, subclinical psychopathy, and subclinical Narcissism are inter-correlated and are part of the “dark triad” construct. The dark triad of personality provides a framework to explore specific traits to predict detrimental and manipulative behavior. It encompasses Narcissism, psychopathy and Machiavellianism into one latent superordinate trait that subsumes all their common characteristics of malevolence [41].

Regardless of the individual scores in manipulators, one of the topics of debate is whether men or women score higher in dark triad variables. Gardner thought, at least initially, that the mothers were most often the manipulators, and many female authors think that fathers are the manipulators. Nevertheless, there is evidence that males score higher in dark triad traits [42,43] and cultural gender roles also influence the expression of dark triad traits [44]. Additionally, we must consider that children can also show dark traits like, Machiavellianism [45,46,47]. 

So far, the relationship between dark traits and the manipulation of children to attack the other partner has not been examined. The purpose of the current research is to determine whether people who are in a process of couple break-up and who are willing to lie in issues concerning their children possess higher levels of the three dimensions that comprise the dark triad. Whether people with higher levels of Machiavellianism, psychopathy, and/or Narcissism (that is, the “dark personality”) will admit they are more capable of deceiving and lying in court is of practical interest. The objective of this study is to build a judicial manipulation scale to measure willingness to lie and use children to harm the other parent that could be used in professional practice. Our main hypotheses are that this scale would be correlated to each of the traits in the dark triad, so that these traits could be used as potential indicators of judicial manipulation during or after child-custody litigation. Dark triad variables are thus expected to be predictors of an individual’s agreement with “dirty” judicial behavior.

## 2. Study 1

### 2.1. Materials and Methods

#### 2.1.1. Participants

We administered questionnaires of the dark triad to 1085 participants (53.5% female), whose mean age was 40.17 (*SD* = 8.7, range 20 to 60). All of them currently had underage children and they had undergone a process of couple break-up within the previous 3 years (which ensured that they understood the problem). On average, parents have had their children when they were 31.12 years-old (*SD* = 6.40). Most parents (63.9%) had one child, while 30.5% had two, 4.4% had three and 1.2% had four or five. The mean age of the children was 9.05 years (*SD* = 5.16, ranging 0 to 17). None of the participants contacted refused to participate.

#### 2.1.2. Materials

Participants answered to the Mach-IV Machiavellianism questionnaire, the narcissistic personality inventory (NPI) Narcissism Scale, Levenson’s Psychopathy Scale, and a questionnaire developed to measure willingness to lie in a court setting in order to gain advantage or harm the partner in a child-custody dispute.

The Mach-IV Scale [48] was used in this study. There are six versions of the Machiavellianism scale, although the most widely used have been the fourth and the fifth versions because the first three versions present interpretation and scoring difficulties. In this work, we decided to use the Mach-IV, as it is still the most widely used and with the most adequate psychometric properties. This version has 20 items (e.g., “Anyone who completely trusts someone is asking for trouble”). Nine items belong to the subgroup of Manipulation Tactics, nine are included in the group of people’s views, and two items are part of the group of Moral Principles. Although the scale is divided into three subgroups, when scoring, the 20 items of the scale are added. It is responded on a 7-point Likert format ranging from 7 (high Machiavellianism) to 1 (low Machiavellianism). Ten of the 20 items that make up the scale are reversely drafted in order to prevent response bias. Several authors [49,50,51,52] have verified the psychometric properties of this scale in Spanish population. Reliability for this scale was modest (α = 0.61), although this is a recurrent issue with Machiavellianism scales [53].

The Narcissistic personality inventory (NPI) [53] is comprised of 40 items (e.g., “I am an extraordinary person”) rated on a 6-point Likert-type response format, ranging from “strongly disagree” (1) to “strongly agree” (6). It measures the following facets of Narcissism: authority (8 items), exhibitionism (7 items), superiority (5 items), entitlement (6 items), exploitation (5 items), self-sufficiency (6 items), and vanity (3 items). The first version created by Raskin [54] had 81 items but later, the authors developed a shorter version, with 40 items [53] which was used in this research. According to its authors, the instrument has a reliability of 0.72. For this work, we used the Spanish version, whose psychometric properties were confirmed by García-Garduño and Cortés-Sotres [55]. For the purpose of this study, we considered Narcissism global score. Its reliability was acceptable (α = 0.81).

Levenson’s primary and secondary psychopathy scales (LPSP) [56] is composed of 26 items (e.g., “Success is based on the survival of the fittest. I am not worried about losers”). The first 16 measure primary psychopathy (callousness, lack of empathy), and the last 10 measure secondary psychopathy (impulsivity). The response form is a 5-point Likert-type ranging from “strongly disagree” (1) to “strongly agree” (5). In this study, we used the aggregated psychopathy score, which showed acceptable reliability (α = 0.86). 

We developed a questionnaire to measure judicial manipulation in custody litigations. It begins with a vignette describing couple break-up and respondents are asked to answer how likely they would be to carry out a list of behaviors during or after a custody dispute. It is divided in two: in the first part, respondents are requested to rate their degree of agreement with 11 items regarding telling lies about the spouse in order to improve their success chances in court and harm their ex-partner. Items are rated on a 4-point Likert-type format ranging from 1 (I would never do this) to 4 (I would surely do this). The second part describes the situation two years after the break-up in which conflict with the ex-partner continues. Participants answered to 7 statements in which the other parent is falsely accused, or which reflect the respondent’s attempts to manipulate the children to harm the other parent, regardless of their children’s welfare. The response system is the same as for the first part. The statements were based on real-case scenarios gathered through professional practice by the authors. The vignettes used can be found in the Appendix A.

#### 2.1.3. Procedure

A total of 26 volunteer surveyors collected data from participants in exchange for course credit using a snowball sampling technique. All participants signed an informed consent to take part in the research and responded anonymously. This research was approved by the Ethics Committee of the University of the corresponding author (project identification code 04/19). It complied with the criteria of Helsinki and with the ethical principles of the American Psychological Association [57] and Helsinki Protocol [58].

#### 2.1.4. Data Analysis

We examined the inter-judge reliability of the items in the judicial manipulation questionnaire calculating the percentage of agreement with the occurrence of each item in the judicial processes of family law. Raters indicated whether each proposed item would occur during a custody dispute judicial trial using a 5-point scale, from “never” to “very often”. We selected the items with higher pair-agreement among people who methodologically acted as experts: one of them was a family law judge, the other a family law prosecutor, and the third was a psychologist specialist in elaboration of expert reports on issues related to family law. To check the soundness of the scale, we performed reliability checks. We also randomly split the sample in two halves to perform an exploratory factor analysis (EFA) with one of the halves (*N* = 545) and a confirmatory factor analysis (CFA) with the other (*N* = 540). For the CFA, as Chi-square is influenced by sample size [59] and model size [60], and both are large in our study, so we relied more on the root mean square error of approximation (RMSEA), the comparative fit index (CFI) and the normed fit index (NFI) to check goodness of fit. Correlations with dark triad variables and a structural equation model (SEM) to predict judicial manipulation form dark traits were also calculated.

### 2.2. Results

First, we calculated the percentages of inter-rater agreement with the likelihood of each of the items that comprised the questionnaire. The selected items met the requirement of inter-expert pair-agreement, and we confirmed that the mean agreement percentage was higher than 90%. Specifically, the mean pair-agreement percentage was 97%: the minimum was 95% and the maximum was 100%. The percentage of global agreement of the comparisons between Expert 1 and Expert 2, Expert 1 and Expert 3, and Expert 2 and Expert 3 was 98%. Therefore, the selected items showed a very high inter-rater agreement.

We checked the adequacy of the judicial manipulation scale devised for this study. The Cronbach’s alpha for the 18 items in the questionnaire was 0.95, showing a good reliability. There were no significant differences in the scale for males and females (Males: *M* = 1.56, *SD* = 0.62; Females: *M* = 1.52, *SD* = 0.58). Next, we carried out a principal components EFA with no rotation and selecting eigenvalues over 1, to check whether the items loaded on to a single factor. We obtained 3 factors, but each item loaded higher on the first factor than on any other. This factor explained 54.82% of the variance with an eigenvalue of 9.87 and item loadings ranging from 0.652 to 0.795. The KMO index (Kaiser–Meyer–Olkin sample adequacy measure) was 0.941, above the recommended value of 0.6, and Bartlett’s sphericity (χ^2^(153) = 7993.268; *p* < 0.001) was significant. The loading of each item on the factor can be seen in Table 1 below. 

We also examined whether this factor could be confirmed through CFA with the second half of the split sample. A single factor solution showed adequate goodness of fit (RMSEA = 0.001 [90% CI: 0.001–0.066]; CFI = 0.99; NFI = 0.99).

Concurrent validity was determined by calculating the correlation between the judicial manipulation scale and the tests that measure the dark triad, as shown in Table 2 below 

We found no significant sex differences for the correlations between dark triad traits and judicial manipulation. There were sex differences for Narcissism (*t*(1083) = 3.72, *p* < 0.001; males: *M* = 3.45, *SD* = 0.42; females: *M* = 3.35 *SD* = 0.47) and psychopathy (*t*(1083) = 5.44, *p* < 0.001; Males: *M* = 3.09, *SD* = 0.58; females: *M* = 2.8 *SD* = 0.65).

**Figure 1:**
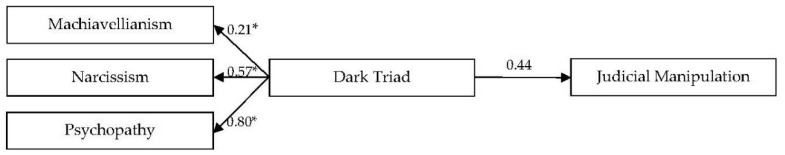
Dark triad as a predictor of judicial manipulation SEM model in Study 1. * *p* < 0.01. χ^2^(2) = 1.46, *p* = 0.48, RMSEA = 0.001 (90% CI: 0.001–0.055), NFI = 0.99, CFI = 0.99).

Figure 1 below shows the relative contribution of each dark trait to the prediction of judicial manipulation. Psychopathy appears as the strongest predictor of all three.

## 3. Study 2

Using a new sample of parents who had undergone a couple break-up and had children, we attempted to replicate the results in Study 1.

### 3.1. Materials and Methods

#### 3.1.1. Participants

We collected data from 249 participants (65.5% female) with a mean age of 43.52 (*SD* = 6.93, range 27 to 62). On average, parents have had their children when they were 33.28 years-old (*SD* = 5.30). Most parents (59.8%) had one child, while 36.1% had two and 4.1% had three. Children mean age was 10.24 (*SD* = 4.90, range 0 to 17). None of the participants contacted refused to participate.

#### 3.1.2. Materials, Procedure and Data Analysis

We used the same questionnaires and procedure used in the first study for the second study except that this time the sample was not split for the factor analyses. Reliability for the scales used was similar to the first study (Mach-IV α = 0.61; NPI α = 0.81 and LPSP α = 0.84).

### 3.2. Results

**Figure 2:**
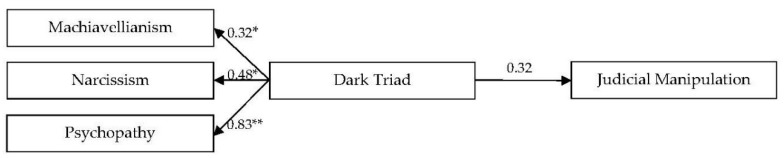
Dark triad as a predictor of judicial manipulation SEM model in Study 2. *p < 0.01, ** *p* < 0.001. χ^2^(2) = 0.32, *p* = 0.98, RMSEA = 0.001 (90% CI: 0.001–0.001), NFI = 0.99, CFI = 0.99).

With this second sample of participants, the judicial manipulation scale showed a Cronbach’s alpha of 0.92. The mean score in the scale in this sample was lower compared to the first study females (males: *M* = 1.08; *SD* = 0.25. females: *M* = 1.18; *SD* = 0.36). The EFA carried out showed a first factor which explained 48.36% of the variance and had an eigenvalue of 8.70, with every single item loading higher on this factor than on any other (loadings from 0.557 to 0.795). The KMO index was 0.816 and Bartlett’s test of sphericity was significant (χ^2^(153) = 3826.798; *p* < 0.01). Item loadings 0.557 to 0.795. The CFA for a single factor solution showed adequate goodness of fit (RMSEA = 0.001 [90% CI: 0.001–0.001]; CFI = 0.99; NFI = 0.99). Table 3 below shows the correlations for variables in the study. There were no sex differences in dark triad scores in this study. Figure 2 shows the SEM model to predict judicial manipulation.

In this second study we found sex differences for narcissism (*t*(247) = 2.23, *p* < 0.05; males: *M* = 3.33, *SD* = 0.40; females: *M* = 3.18 *SD* = 0.53) and judicial manipulation (*t*(247) = 2.27, *p* < 0.05; males: *M* = 1.08, *SD* = 0.25; females: *M* = 1.18 *SD* = 0.36). Figure 2 shows the SEM model to predict judicial manipulation. As in Study 1, psychopathy is the strongest predictor of judicial manipulation. 

## 4. Discussion

There are a number of parents who are willing to lie in a judicial process and even manipulate their children in order to harm the other parent. These parents are more Machiavellian, Narcissistic, and they have a subclinical psychopathic personality. The scale developed in this study, in which parents are asked whether they would be willing to deceive and lie to the court to achieve their goals—which is often a revenge on the parent who broke up the relationship—comprises both the willingness to provide false information about the other parent and manipulating children. The scale shows a high reliability and loads primarily on to a single factor in EFA and is confirmed by CFA. This scale correlates significantly with the variables that make up the dark triad. Therefore, the judicial manipulation scale makes an important contribution to the scientific advancement in the field of psychological-forensic assessment and, consequently, to help the courts to determine which parent should be assigned as the children’s guardian, and what kind of visiting regime should be established for the other parent. Its primary use would be in research of which variables predict judicial manipulation. We showed that dark triad traits are adequate indicators of judicial manipulation, and the scale can serve as a criterion to check other possible indicators.

As with other essential premises that support the PAS ideas of Gardner [13,14], the results of this study confirm that parents’ possible manipulative behaviors during and after the family break-up are explained by specific personality traits of the parents, rather than by a parental alienation syndrome as Gardner [1,2,3,4] stated. Rather than evaluating custody disputes drawing from models like the PAS, we propose the dark traits provide a more promising framework. Thus, dark traits of personality are a good indicator of parent’s manipulative behaviors and lying to attack the other parent. Variables in the dark triad are significantly correlated with the scale developed for this study. Psychopathy seems to be the best predictor of judicial manipulation among dark traits and it also shows a lack of sensitivity to socially desirable responding. Additionally, contrary to other authors [42,43] we found scarce evidence for sex differences in dark traits.

We think that an important aspect in this research is determining which variables predict whether an individual is a judicial manipulator, which has a direct use in practice, and the vignettes used in this study stand as a useful tool for examining other possible indicators of manipulative behavior in a court setting.

This research presents some limitations that should be overcome in future research: we did not get the chance to measure the responses of parents who were currently litigating for the custody of their children as a validation criterion of the scale. In addition, its self-report and correlational nature advises for caution when drawing conclusions from these results. 

A relevant issue when exploring dark traits, manipulation or any attitude or behavior that may be socially questionable is whether it is subject to socially desirable responding. Although the participants answered anonymously, there may still have been some social desirability response bias. We did not use a measure of socially desirable responding to control for possible biases in responses and this is another limitation in our research. However, evidence shows that psychopathy is negatively associated with social desirability, suggesting that individuals who score high in psychopathy may not value social acceptance [61]. Other research results [62] show that individuals high in Machiavellianism and Psychopathy are more unconcerned with social desirability. Psychopathy is associated with a diminished sense of morality and is associated with a worse understanding of what is socially desirable. Neither Machiavellianism nor Narcissism show a consistent association with social normativity. Lastly, Machiavellian individuals seem to care more about their own goals rather than about social impressions, and the more antagonistic a dark trait is, the less important is for the individual to respond in a socially desirable way [63]. As for gender differences, females score higher in social desirability, and it has been argued that this may partially explain why they score lower in dark triad traits [64]. Nevertheless, motivational goals and socially normative biases in parents’ responses may certainly obscure the relationship between dark trait indicators and judicial manipulation, and extensive research is needed to check which indicators provide less biased responses. 

However, being able to resort to indicators which are not domain-specific or directly related to gaining advantage in a judicial setting may provide a useful tool for professional practice. We have examined just the three most prominent dark traits in literature, although there are many other variables that could be considered as indicators of judicial manipulation.

## 5. Conclusions

This research suggests a better framework than PAS to understand parents’ potential proneness to manipulate or lie in court during or after a custody litigation. Variables in the dark triad appear to be adequate predictors of willingness to lie or manipulate in a court setting and these results call in for further research to determine additional traits useful to determine judicial manipulators. The questionnaire developed in this study is of practical use as it provides a criterion to test which personality traits can be indicators of foul play in child custody disputes.

## Figures and Tables

**Table 1:** Factor loadings for the judicial manipulation scale.

Scale Item:	Loading
My partner regularly insulted me and despised me	0.726
My partner physically assaulted me	0.763
My partner treated my children badly, despised them and insulted them	0.764
My partner hit my children and sometimes physically hurt them	0.749
My partner earns much more money than he/she declares, and should pay a high alimony for the children	0.664
The children do not love him/her, they only want to be with me	0.784
My partner never bothered with the children’s food or cleanliness	0.751
My partner knows nothing about the children’s school progress, only I deal with this	0.695
My partner’s family gets on better with me than with their son/daughter	0.719
My partner is an aggressive person with whom it is difficult to talk	0.795
Meeting my partner was a big mistake in my life	0.675
Stop paying the children’s alimony that I must pay	0.731
Prevent my partner’s contact with the children when it is legally stipulated	0.779
Instill animosity and even hatred towards the other parent in the children through my direct and indirect comments	0.772
Convince the children that they should tell the court technicians that the other parent treats them badly psychologically (contempt, insults)	0.787
Convince the children that they should tell the court technicians that the other parent treats them badly physically (hits them and attacks them)	0.764
Attack the children physically, as I am convinced that this is the best way to attack the other parent	0.652
Continually file complaints against the other parent (false allegations), for any reason, just so he/she will lose prestige judicially	0.737

**Table 2:** Correlations between judicial manipulation and the dark triad by sex in Study 1.

	Machiave Llianism	Narcissism	Psychopathy	Judicial Manipulation
Machiavellianism		0.13 *	0.15 **	0.17 **
Narcissism	0.12 *		0.41 **	0.23 **
Psychopathy	0.17 **	0.50 **		0.37 **
Judicial Manipulation	0.07	0.26 **	0.34 **	

* *p* < 0.01; ** *p* < 0.001. Upper half: female (N = 581); lower half: male (N = 504).

**Table 3:** Correlations between judicial manipulation and the dark triad traits by sex in Study 2.

	Machiave Llianism	Narcissism	Psychopathy	Judicial Manipulation
Machiavellianism		0.15 *	0.30 **	0.17 **
Narcissism	0.19		0.38 **	0.27 **
Psychopathy	0.22 *	0.42 **		0.43 **
Judicial Manipulation	−0.10	−0.20	−0.09	

* *p* < 0.05; ** *p* < 0.001. Upper half: female (N =163); lower half: male (N = 86).

## Appendix A. Vignette

Please try to imagine yourself in the following situation:

The arguments with your partner are becoming worse, and you finally decide to break up. Your partner does not object to this, but he/she does have objections about your two children, aged four and nine years. You ask people who understand the judicial process for advice, and they recommend that, as above all, your partner wants to be with the children, what you should do in the trial is to issue a series of statements—no matter if they are true. This person explains that everyone does this, and you go to trial to beat the other party, and you will not regret it.

Next, please read the statements that this person proposes you to make. You rate your answers on the scale to the right, marking “1” if you would never do that, “2” if you think that you would not do it, “3” if you might do it, and “4” if you would surely do it. We remind you that this information is anonymous, so we ask for complete sincerity:

	1. I would never do it	2. I don’t think I would do it	3. I might do it	4. I would surely do it
1. My partner regularly insulted me and despised me	1	2	3	4
2. My partner physically assaulted me	1	2	3	4
3. My partner treated my children badly, despised them and insulted them	1	2	3	4
4. My partner hit my children and sometimes physically hurt them	1	2	3	4
5. My partner earns much more money than he/she declares, and should pay a high alimony for the children	1	2	3	4
6. The children do not love him, they only want to be with me	1	2	3	4
7. My partner never bothered with the children’s food or cleanliness	1	2	3	4
8. My partner knows nothing about the children’s school progress, only I deal with this	1	2	3	4
9. My partner’s family gets on better with me than with their son/daughter	1	2	3	4
10. My partner is an aggressive person with whom it is difficult to talk	1	2	3	4
11. Meeting my partner was a big mistake in my life	1	2	3	4

Two years have gone by and the relationship with the other parent is bad. There are constant problems with anything that involves the children. Now your partner has denounced you in Court, you think unfairly. Again, your acquaintance advises you, and in this case, he/she proposes a number of actions. Now we ask you to answer the extent to which you would carry out such actions. Again, we remind you that this information is anonymous, so please respond sincerely:

	1. I would never do it	2. I don’t think I would do it	3. I might do it	4. I would surely do it
1. Stop paying the children’s alimony that I must pay	1	2	3	4
2. Prevent my partner’s contact with the children when it is legally stipulated	1	2	3	4
3. Instill animosity and even hatred towards the other parent in the children through my direct and indirect comments	1	2	3	4
4. Convince the children that they should tell the Court technicians that the other parent treats them badly psychologically (contempt, insults)	1	2	3	4
5. nvince the children that they should tell the Court technicians that the other parent treats them badly physically (hits them and attacks them)	1	2	3	4
6. ttack the children physically, as I am convinced that this is the best way to attack the other parent	1	2	3	4
7. Continually file complaints against the other parent (false allegations), for any reason, just so he/she will lose prestige judicially	1	2	3	4
